# Deconvolution of autoencoders to learn biological regulatory modules from single cell mRNA sequencing data

**DOI:** 10.1186/s12859-019-2952-9

**Published:** 2019-07-08

**Authors:** Savvas Kinalis, Finn Cilius Nielsen, Ole Winther, Frederik Otzen Bagger

**Affiliations:** 1 Centre for Genomic Medicine Rigshospitalet, University of Copenhagen, Copenhagen, Denmark; 2 Section for Cognitive Systems Department of Applied Mathematics and Computer Science, Technical University of Denmark, Lyngby, Denmark; 3 Bioinformatics Centre Department of Biology, University of Copenhagen, Copenhagen, Denmark; 4 University Children’s Hospital Basel and Department of Biomedicine, University of Basel, Basel, Switzerland; 5 Swiss Institute of Bioinformatics, Basel, Switzerland

**Keywords:** Interpretable machine learning, Deep learning, Neural networks, Manifold learning, Expression profiles, Single-cell RNA-sequencing, Gene set enrichment analysis, Functional analysis, Biological pathway analysis

## Abstract

**Background:**

Unsupervised machine learning methods (deep learning) have shown their usefulness with noisy single cell mRNA-sequencing data (scRNA-seq), where the models generalize well, despite the zero-inflation of the data. A class of neural networks, namely autoencoders, has been useful for denoising of single cell data, imputation of missing values and dimensionality reduction.

**Results:**

Here, we present a striking feature with the potential to greatly increase the usability of autoencoders: With specialized training, the autoencoder is not only able to generalize over the data, but also to tease apart biologically meaningful modules, which we found encoded in the representation layer of the network. Our model can, from scRNA-seq data, delineate biological meaningful modules that govern a dataset, as well as give information as to which modules are active in each single cell. Importantly, most of these modules can be explained by known biological functions, as provided by the Hallmark gene sets.

**Conclusions:**

We discover that tailored training of an autoencoder makes it possible to deconvolute biological modules inherent in the data, without any assumptions. By comparisons with gene signatures of canonical pathways we see that the modules are directly interpretable. The scope of this discovery has important implications, as it makes it possible to outline the drivers behind a given effect of a cell. In comparison with other dimensionality reduction methods, or supervised models for classification, our approach has the benefit of both handling well the zero-inflated nature of scRNA-seq, and validating that the model captures relevant information, by establishing a link between input and decoded data. In perspective, our model in combination with clustering methods is able to provide information about which subtype a given single cell belongs to, as well as which biological functions determine that membership.

**Electronic supplementary material:**

The online version of this article (10.1186/s12859-019-2952-9) contains supplementary material, which is available to authorized users.

## Background

Recent upsurge of data generated by mRNA sequencing at the single cell level (scRNA-seq) have helped to address a number of scientific questions and have also revealed new challenges. It allows researchers to look into gene expression levels of a specific cell, rather than the aggregated levels that came with “bulk” RNA sequencing, and create fine molecular profiles of tissues, that are particularly important for insights into the dynamics and function of more heterogeneous tissues, such as cancer tissues.

Using scRNA-seq it has been possible to delineate cellular populations in an unbiased manner from several healthy [1–4] and diseased tissue [5, 6], and a large number of new methods have addressed the new computational and analytical challenges with this data type [7–9].

Modeling of the scRNA-seq data is challenging because relevant and often categorical biological signal is usually intertwined with dynamical biological processes (i.e. cell cycle, maturation, differentiation or metabolic activity) as well as technical sources of variation (i.e. PCR amplification, “dropout” events, sequencing or library preparation variation tissue dissociation and many parameters related to laboratory protocol).

Recently, there have been several excellent attempts to model scRNA-seq data using prior knowledge on specific sources of variation [10, 11]. In this study, however, our aim is to extract biological information from a class of more general, non-linear models, that can assimilate the information of the manifold shaped by the single-cell expression profiles.

Artificial neural networks (NN) have proven flexible and demonstrated representational power and state of the art results in many applications (i.e. skin cancer classification [12], retinal disease diagnosis [13], protein folding [14, 15]). In addition, recent advancements in the development of software frameworks that efficiently exploit computing resources, mostly by parallel processing on GPU, render the definition, implementation and training of a NN quite straightforward.

We hypothesise that simple NN layouts and stringent training will make deconvolution possible and tease apart biological signal from heterogeneous cellular populations. We believe that the distributed nature of NN models bears the potential of encapsulating, rather than smoothing over or regressing out sources of variation, both biological and technical.

In this study we applied autoencoder neural networks [16], unsupervised machine learning methods, to scRNA-seq expression counts. This class of models are used as a manifold learning technique and are able to efficiently capture the underlying signal even when the input is perturbed or zeroed out [17], which is particularly appealing for an application to scRNA-seq data. Variants of autoencoders have been successfully applied to scRNA-seq data before, for dimensionality reduction, denoising and imputation of missing values (see [18–26] for a complete list of studies).

Here, we will make use of a simple autoencoder architecture and apply methods from the computer graphics community, known as saliency maps [27], aiming to deconvolute what the latent representation of the model captures, and to interpret it in terms of biological pathways.

## Results

**Fig. 1:**
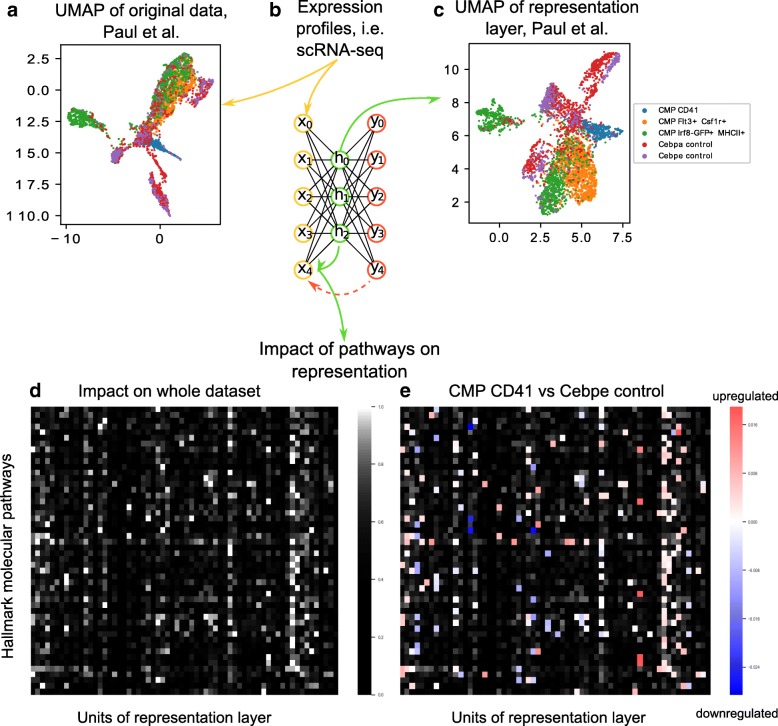
General overview of our approach. Expression data act as input to the autoencoder (**b**) which models the data. The model’s representation of the data set can be visualized by a dimensionality reduction plot (**c**). The impact of gene sets of interest to our representation method can be visualized, either for the whole data set (**d**) or for a comparison between two groups of cells (**e**). **b**: A general outlook of an autoencoder artificial neural network. The autoencoder shown has an input, a hidden and an output layer, but it is common that it contains more hidden layers. Usually the hidden layer in the middle of the network acts as the representation layer, which contains the compressed information of the original data. The representation is decompressed in the output layer, where the input is recreated with some accuracy. **a** & **c**: Uniform Manifold Approximation and Projection (UMAP) of Paul et al. The UMAP of the original input data is visualized on (**a**) and UMAP of the evaluation of the representation layer, after training is done, is visualized on (**c**). We can see that the neighboring structure of the original input data is retained in the representation layer. **d** & **e**: Heatmaps of the impact of the Hallmark molecular pathways on the representation layer of the autoencoder trained on Paul et al. The impact is computed via saliency maps (see Methods section). To enhance visual clarity, only the high impact pathways are visualized. We plot the impact of the gene signatures for the whole dataset (**d**) and for the comparison between two groups of the dataset, CMP CD41 and Cebpe control, which also includes differentiated cells (**e**). The comparison is done by subtracting the impact of the hallmark pathways of one group versus the other. The difference in impact is overlaid on the “general” heatmap (**d**)

A simple autoencoder with three layers (input layer, a hidden or representation layer and an output layer) can be seen on Fig. 1b. Each layer consists of a number of units, corresponding to its dimensionality. Briefly, an autoencoder is trained to learn how to recreate the input in an output layer. The challenge is to first compress the input to the internal representation (can be viewed as the “encoding” process) and then decompressing onto the output layer. In essence a nonlinear dimensionality reduction is performed, meaning that we are able to inspect the original dataset in a manifold of lower dimension. Furthermore, from the output we are able to assess whether a sufficiently complex representation was made (by evaluating the information loss during compression from input to output).

In this study we trained an autoencoder with a soft orthogonality constraint on the representation layer alongside a Poisson loss function. The orthogonality constraint pushes the representation layer to contain information that is disentangled between units.

We applied our model to the scRNA-seq dataset produced by Paul et al. [2]. With a suitable learning rate we were able to train the model directly on the read count data (without log normalization or preprocessing). Fig. 1a and c show the 2-dimensional Uniform Manifold Approximation and Projection (UMAP) [28] embedding of Paul et al. for the original input and the representation layer, after training is done, respectively. For the UMAP of the representation layer, we evaluate each single cell through the encoding part of our network and keep the values of the lower-dimensional representation. We then apply UMAP on those representation values.

The embedding and the value of the test loss function after training are convincing regarding the successful application of the autoencoder as a robust dimensionality reduction tool that handles dropouts well. Our aim is to deconvolute the resulting model and establish a link between the representation layer of our model and biological function. We evaluate the impact of gene sets on the representation layer of the network by the use of saliency maps. Strikingly, we find that each hidden unit in the distributed model appears to model a distinct term or modality in the data. We saw less entanglement or spillover between nodes, than we expected given the colinearity of gene expression data. It appears that the division of labour is well-defined, and may have intelligible interpretation. In Fig. 1d we visualize the impact of each of the hallmark molecular pathways [29] to our hidden units in a heatmap (a zoomed in version of Fig. 1d and e can be found as Additional file 1: Figure S1). This way we can identify pathways with high impact on hidden units.

We also investigate the difference in impact between two known cellular populations displaying only the high impact pathways, that are less likely to model noise terms. In Fig. 1e we visualize the difference in impact for ‘CMP CD41’ and ‘Cebpe control’ of the Paul et al. dataset. From the latter heatmap we can identify pathways that behave differently between the two groups under investigation, in terms of the impact of that signature. The selected populations are Common Myeloid Progenitor cells (CMP), and a full haematopoietic background, which also contains mature and differentiating cells, as reference. The direction of change in hidden units that could signify stemness or progenitor states are up in CMP, i.e. WNT-{beta}catenin-signaling, described as key stemness factor [30], and DNA repair and hypoxia, both associated with stemness [31, 32]. Relative to the control, the CMPs show less activity in pathways that could be associated with differentiation, division and maturation, in terms like mitotic spindle, Apical changes and Hedgehog signaling [33].

In order to validate that each identified module corresponds to a functional category, we applied our model to Velten et al. [1], where we have detailed fluorescence-activated cell sorting (FACS) data for each cell, effectively describing their cellular identity, in terms of immunostaining. This dataset consists of human hematopoietic stem cells. The UMAP embedding of that dataset for original input data and representation data is displayed on Additional file 2: Figure S2. We show that the neighboring structure of the single cells is, again, retained in the lower dimensional representation layer. In this scenario we followed a case specific approach and investigated the impact of hematopoiesis related signatures, derived from DMAP [34] on the representation layer. In Additional file 3: Figure S3 we show six heatmaps, one for each progenitor state, as derived by FACS. The progenitor states are defined as shown in Table 1. In the heatmap, haematopoietic signatures are shown as rows and hidden units as columns. Colours are based on the impact of the genes in the signatures, vailing low impact nodes.

**Table 1:** Definition of cell types from FACS markers for Velten et al. scRNA-seq data

	MEP	CMP	GMP	HSC	MPP	MLP
SSC	low	low	low	low	low	low
FCS	mid	mid	mid	mid	mid	mid
lin	low	low	low	low	low	low
CD38	high	high	high	low	low	low
CD34	high	high	high	high	high	high
CD10	low	low	low			high
CD90				high	low	
CD135	low	high	high			
CD45RA	low		high	low	low	high

Defining cell types from FACS markers in data from Velten et al., as suggested by the authors, but with hard gates. High is top 50% of the cells expressing that marker, low is 50% lowest expressed, and mid is the 2nd and 3rd quartile. *HSC* hematopoietic stem cell, *MPP* multipotential progenitors, *CMP* common myeloid progenitor cell, *GMP* granulocyte monocyte progenitors, *MEP* megakaryocyte-erythroid progenitor cell

CMP cells, as identified by FACS (please see Table 1 for definitions of cell types)), clearly elicited activity in hidden neurons responsible for modelling CMP signature genes, as identified by differential expression by gene expression data from the well-annotated DMAP study, as well as progenitor cells to CMP, like MEP, GRN. All cells in the study are HSC and progenitor cells, and HCS signature is also active for all but lymphoid cells. GMPs are closely related to CMPs, and show similar activity, but with more activity in GMP signature. The MEP signature is active in all erythroid cells, which are also progenitors thereof.

**Fig. 2:**
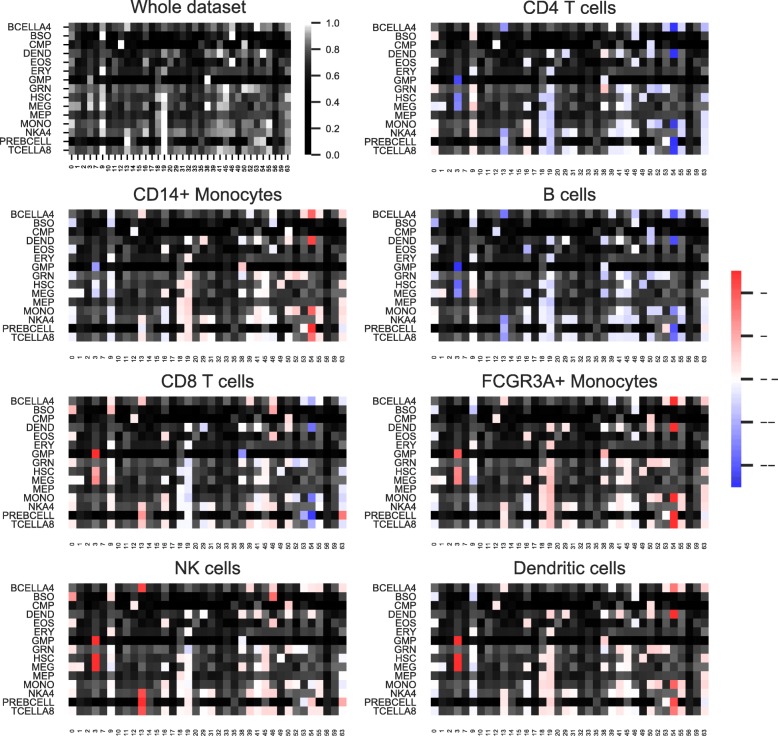
Impact of hematopoietic signatures on the representation layer of our autoencoder Impact of hematopoietic signatures (rows) on the representation layer (columns) of the autoencoder trained on PBMC data. The hematopoietic signatures are derived from the DMAP study. To enhance visual clarity, only the high impact pathways are visualized. The top-left heat map corresponds to all the cells. The rest of the heat maps correspond to a summary of cells in each cellular population of the study, as clustered and labeled by Seurat. Row names correspond to cell types categories, or to DMAP labels for sub-classification, where TCELL8A is CD4 + CD45RA-CD62L+ T-cells and BCELL4 is CD19 + lgD-CD27+ B-cells, respectively. Not shown are Seurat predicted clusters on Megakaryocytes cells (< 1% in human PBMC)

We included a further validation step by applying our model to a dataset of Peripheral Blood Mononuclear Cells (PBMC) [35]. In order to compare the cell type signatures that are active in hidden units in our model with cell type label predicted by Seurat we summarised the back-propagated activity of the Seurat clusters (Fig. 2) in our model of the PBMC data. For each of the clusters of cells it is clear that the same cell type is active in the representation layer, as predicted by Seurat, except for CD8 T-cells which does not seem to either have diffuse profile or not to match any T-cell signatures from DMAP (data not shown). For the remaining signatures there is a high overlap, and whereas B- and T-cells are expected to be more similar than eg. Monocytes [34]. Seurat predicted T-Cells are more intense in B-cell signature than the B-cells, which may be due to incomplete set of signatures to describe the data. Only on unit 45–46 there seem to be a dedicated signal for these B-cells, assuming that Seurat has correctly labeled all the cells. NK cells show similarity with a number of cells, but are unique in having a clear activity in NK signatures in a hidden unit 13. The difference in the two types of monocytes can be seen in the activity in signatures of progenitor states, thus suggesting a development between the two, which is confirmed by known FACS panels [34].

We tested the output representation of the model by comparing the clustering of our model against the popular Seurat method [36] and clustering on the raw input. We performed 20 iterations of k-means clustering both on the original input and the representation of our model and compared with the output of the clustering from the Seurat method. To perform this comparison we matched the labels of the clusterings to the labels produced by the Seurat method and computed the normalized mutual information for all possible comparisons. The results found show that all three methods have similar clustering output on the PBMC dataset; original vs representation: 0.780, representation vs Seurat: 0.761, original vs Seurat: 0.785.

In order to ascertain the contribution of the orthogonality criterion, we measured the L2 norm of the singular values of the representation of the input, with and without the orthogonality criterion. In our experiments, the orthogonality criterion improved the aforementioned norm, for varying orthogonality coefficients. The measured norm was reduced by 78.9% more per epoch when the best orthogonality constraint was used, compared to when no orthogonality constraint was used.

## Discussion

The autoencoder model we trained is simple, robust and small enough to run on a regular computer. Adding to the model’s simplicity, we are able to feed raw expression data to the model, entirely skipping normalization and transformation processes which usually precede common dimensionality reduction techniques. In this study we applied the model to scRNA-seq expression data, but exactly the same approach could be followed with other types of expression data, i.e. sequencing or microarray of bulk mRNA, epigenetic marks or mutations, if only the space can be reduced sufficiently to be deciphered through signatures of genes or positions. The good adaptation to sparse data with random dropouts make the system, and future developments hereof, very well suited for scRNA-seq, which will only become more important in the near future.

With the usage of saliency maps, we attempt to establish the critical link between the mathematical models that describe an expression dataset well and the biological functions that are active in the dataset. The orthogonality requirement is key to achieve this. We added the soft orthogonality criterion in the loss function, as an attempt to deconvolute the highly correlated biological signal, and so that each of the hidden units correspond in essence to one dimension of the representation layer. The effect of the orthogonality constraint could be further quantified by the L2 norm of the singular values of the representation of the input and was indeed shown to improve the reduction of that norm per epoch. Further to saliency maps a number of alternative visualisation methods exists, that may also be able to recapture biological meaningful representations for models trained in a similar constrained manner [37].

Case specific gene sets can be chosen by the researcher for specific interpretations of single cells. Oversaturation of the signature space or testing of correlating signatures should carry smaller risk of misinterpretation; selection of signatures does not change the model, nor requires retraining, and the effect is apparent from a simple heatmap. When more and better annotated scRNA-seq data is available in the future, it will be shown how this method can assist in deciphering, not only the status or class of a single cell in a population, but also its total activation within several categories. This is particularly important for continuous cellular spaces, or to disentangle the contribution of cellular state, cellular type or cellular environment.

We used UMAP as a visualization technique for single cell data due to its efficiency, computational and mathematical rigor advantages over similar commonly used methods, i.e. PCA, t-SNE [38]. UMAP focuses on displaying the neighboring structure of the multidimensional manifold in few dimensions. As we’ve seen in Fig. 1, the single cells retain the same neighbors in the UMAP of the original data and the UMAP of the representation. However, that should not be the sole criterion when judging the efficacy of a method. To this point, we would like to advise to be cautious when interpreting the 2-dimensional representations of multidimensional data. The original dataset lies on a multidimensional space and this should not be neglected when inferring biological relations (Additional file 2: Figure S2 provides additional visual explanation to this point).

Our model differs from popular existing methods, i.e. Seurat, SC3 [39], in the identification of gene modules of interest. Although the aforementioned methods exhibit better clustering performance than our model, partly due to the application of graph-based methods, the marker gene detection in both methods relies upon identification of differentially expressed genes, via simple statistical tests of multiple regression. These tests may be suitable for identification of marker genes of simple traits, but for more complex datasets with added heterogeneity like cancer, this approach may prove insufficient. A nonlinear neural network is suitable for pattern recognition in complex data and through guided backpropagation of the signal (as performed with saliency maps), we can identify the most important input features (genes) that affect the formation of those patterns. This is a clear prospective advantage of our approach compared to both Seurat and SC3, a more accurate link to the complex biology that is present in a dataset and this advantage will manifest itself in greater scale as the size of the gathered datasets increases. Furthermore, our approach doesn’t require any particular pre-processing, which is always a problematic component, as the separation of analysis and preprocessing (which may have severe implications on the results) can lead to investigation of artifacts.

When comparing results from our model on PBMC data with output from popular single cell analysis suite Seurat we find that we can largely recapture the labels of the clusters predicted by Seurat (PBMC is the dataset in Seurat tutorial, and thus well tested by the authors). We see also that there are overlaps of back-propagated activity between the cell types, and it appears that the activity corresponding to Seurat labels, are mainly those that are uniquely active for one cluster. This fits well with our biological understanding of many shared functionalities (especially in the related PBMCs) between cells, but where some specific processes are unique. In this manner e.g. the NK signatures are active in a dedicated hidden unit overlapping an NK signature. This same hidden unit resembles activity for B- and T-Cells, but B- and T-cells have little activity in that same hidden unit; their identity is signified by another hidden unit. Thus, our questions, in the form of back-propagation to genetic signatures, may not be precise enough to yield unique closed-form answers about the biology represented in the model. It is possible that a complete deconvolution of a large single cell dataset, like the Human Cell Atlas [4], will enable us to uncover, using our model, not only cell types but at the same time biological programs and shared function. This is perfectly possible, since our approach of deconvolution of the model, does not affect the model; different types of signatures can be tested, to pinpoint the identity of each hidden unit, leaving a reduced representation of the data, which can be used both to explain each cell, or cluster of cells, and predict identity or function of future cells.

We believe that application of our model to a plethora of datasets, can lead to synthesis of a fixed feature extractor model for expression data. A fixed feature extractor acts as a pre-trained model that can capture meaningful representations for new, diverse inputs (see [40] for more information on feature extractors). In the future we aim to build on top of our model to create a “universal expression model” that identifies most of the wanted biological relationships of a new dataset. By applying that universal model to a new dataset we will be able to quickly annotate it on various effects, as well as extract information on biological differences on distinct phenotypes of the dataset. This would be a big step forward in our understanding of the biology behind the large expression datasets gathered daily.

## Conclusions

We present an implementation of autoencoders, with an orthogonality constraint on the representation layer, that we apply on scRNA-seq data. We find that the model handles well the noise and dropout level in the data, and are able to recapitulate the original neighborhood structure in the output. By the use of saliency maps we discovered that each hidden unit in the model represent a well-defined module. These modules correspond to a large extent to activity in gene signatures of biological pathways, and we show for three datasets, of different single cell sequencing protocols, that this gives a precise description of the biological phenotype. We believe that our discovery bears the potential for a holistic analysis through autoencoders, where both normalisation, imputation of random dropouts, and analysis can be performed in a single operation.

## Methods

We trained an autoencoder with 2 layers for encoding and 2 for decoding, with dimensions 128, 64 and 128 for the hidden layers. The size of the representation layer was chosen to slightly exceed the number of gene sets under investigation, in our case the hallmark molecular pathways. We limited the input dataset to the genes that were present in the signatures, for faster training and memory fit. The nonlinearity of the encoding and decoding layers was chosen to be the SoftPlus nonlinearity [41]. The weights were initialized with Xavier initialization [42] and the biases with a small constant. The model was trained with a Poisson negative log-likelihood loss function, to account for the fact that RNA-sequencing expression levels are count data. We have previously seen that this generic loss function trains well in scRNA-seq data [21] and it fits the purpose of our current study to provide a general use framework for the identification of biological information from neural network models. Recent studies account for dropouts with specific modeling choices [10], however, this kind of model should always be applied with caution, depending on the underlying zero generating process [43]. Thus the loss function with the added soft orthogonality constraint looks like that:

Loss = mean(x - y * log(x + ε)) + λ * L2_norm(I - WW^T^) (eq.1).

where x is the input, y is the reconstructed input; y = decode(encode(x)), ε is a very small constant, λ is a hyperparameter that determines the impact of the orthogonality constraint, W is the weight matrix of the final encoding layer, W^T^ the transpose matrix of W and I-WW^T^ is the orthogonality constraint.

As opposed to other applications of neural networks to scRNA-seq, we decided to not train with mini-batches, since, due to the nature of single cell data, our aim was to distinguish fine differences between samples. In this particular setting, a mini-batch would push the model towards over-generalization, as beautifully outlined by Li et al. in a visual comparison of the effects of mini-batch size on the loss function optimization process [44].

We chose Nesterov accelerated gradient [45] technique for loss function optimization, that has been shown to outperform and be more stable than ADAM [46], which reputedly works well with sparse data. Hyperparameter optimization was performed with grid search. The model stopped training when the loss in the test set would stop improving for 10 epochs. Training speed is affected negatively by the selection of batch size of 1. Using a standard personal computer with GPU for these experiments the time needed to train was: PBMC: 15.4 min for 70 epochs for input matrix of size (2638, 3009); Paul et al.: 38 min for 310 epochs for input matrix of size (4180, 2560); Velten et al.: 3.5 h for 600 epochs for input matrix of size (1401, 3331). The model was implemented in Python v.3.6.5 scripting language (https://www.python.org/), using the PyTorch v.1.0.0 deep learning framework [47]. The code is available on gitlab: https://github.com/cphgeno/expression_saliency.

The idea behind vanilla saliency maps in deep learning is rather intuitive. We compute the gradient of the representation units with respect to the gene expression input, by testing each representation unit in isolation. That is, we consider that only one representation unit has positive gradient equal to one and the rest have gradient 0, and we let the gradient backpropagate through the network. This way we can see how the representation is affected by small changes in the gene expression levels, or in other words, the impact that each gene has on each representation unit. In our study we compute the guided backpropagation saliency maps, that has shown more clear results [48]. The difference is that only positive gradients flow back to the network, the negative gradients are clipped.

In order to compute the impact of a gene set to each hidden unit, we simply take the arithmetic mean of the impact of the genes in the set. The resulting pathway impact scores are min-max scaled to the range [0, 1]. In the comparison scenario, the impact scores of the cells to compare are subtracted and then scaled. The scaling is now performed by division with the maximum value of the difference in impact scores, so the final pathways impact scores fall in the range [− 1, 1]. Hidden units with zero impact for all pathways under investigation were omitted from the analysis. In this manner we can evaluate the impact of custom gene sets on the representation, as we did here with the hallmark molecular pathways [29] and hematopoietic signatures on Paul et al. and Velten et al. respectively.

The algorithm can be described as follows:

- Train autoencoder neural network, via optimization of loss function (eq.1).
- Pass expression matrix X through autoencoder and plot UMAP of computed representation layer; UMAP(encode(X)).
- For the computation of the impact that a gene set has on the representation layer:
  - Compute the representation layer of an input of C cells under investigation.
  - For each element of the representation layer.
    - Compute the absolute value of the guided saliency (for all C cells).
    - For each input variable (gene) compute the mean saliency, among the C cells.
    - Average previously computed mean saliencies over the genes contained in the gene set.

Hematopoietic signatures were derived from DMAP normalised and processed data (no longer available via Broade Institue web portal. Please find in project git repository), performing differential analysis with limma [49] from R bioconductor in a one-against-others comparison. For validation of which signatures are active a subset of cells was used to represent each population.

## Additional files

Additional file 1: **Figure S1.** Impact of the Hallmark molecular pathways on the representation layer of our autoencoder. Zoomed in version of the heatmaps of the impact of the Hallmark molecular pathways on the representation layer of the autoencoder trained on Paul et al. The impact is computed via saliency maps (see Methods section for more information). To enhance visual clarity, only the high impact pathways are visualized. We plot the impact of the gene signatures for the whole dataset (d) and for the comparison between two groups of the dataset, CMP CD41 and Cebpe control, which also includes differentiated cells (e). The comparison is done by subtracting the impact of the hallmark pathways of one group versus the other. The difference in impact is overlaid on the “general” heatmap (d). (EPS 814 kb)

Additional file 2: **Figure S2.** UMAP of different representations of our autoencoder vs original data. UMAP of original data (top left) and representation layer of the autoencoder for the Velten et al. data set. The clusters of the Velten et al. data set are taken from the Bloodspot database [50]. The representation layer is visualized for varying number of training epochs; after no training (bottom left), training for 10 epochs (bottom right) and after training is done for epoch 1990 (top right). Here we want to illustrate that the 2 dimensional visualizations of a multidimensional dataset can be deceiving. A nice visualization on its own does not qualify as a metric of a well-trained model. (EPS 120 kb)

Additional file 3: **Figure S3.** Impact of hematopoietic signatures on the representation layer of our autoencoder, for model trained on Velten et al. dataset. On the top right we visualize a model of the hematopoietic system. The rest of the plots depict heatmaps of the absolute values of the impact of hematopoietic signatures (rows) on the representation layer (columns) of the autoencoder trained on Velten et al. The hematopoietic signatures are derived from the DMAP study. To enhance visual clarity, only the high impact pathways are visualized. The top left heatmap corresponds to all the cells. The six bottom heatmaps correspond to each cellular population of the study, as defined by the FACS profile. (EPS 196 kb)

## Data Availability

The code is available on gitlab: https://gitlab.com/cphgeno/expression_saliency. Datasets analysed during this study are included in the published articles of Paul et al. and Velten et al. with GEO accession numbers GSE72857 and GSE75478, respectively. PBMC data were downloaded from the Seurat package: https://satijalab.org/seurat/pbmc3k_tutorial.html.

## Abbreviations

CMP: Common myeloid progenitor cell
FACS: Fluorescence-activated cell sorting
GMP: Granulocyte monocyte progenitors
HSC: Hematopoietic stem cell
MEP: Megakaryocyte-erythroid progenitor cell
MPP: Multipotential progenitors
NN: Artificial neural networks
PBMC: Peripheral blood mononuclear cells
scRNA-seq: Single cell mRNA-sequencing data
UMAP: Uniform manifold approximation and projection
